# Melatonin and Structurally-Related Compounds Protect Synaptosomal Membranes from Free Radical Damage

**DOI:** 10.3390/ijms11010312

**Published:** 2010-01-21

**Authors:** Sergio Millán-Plano, Eduardo Piedrafita, Francisco J. Miana-Mena, Lorena Fuentes-Broto, Enrique Martínez-Ballarín, Laura López-Pingarrón, María A. Sáenz, Joaquín J. García

**Affiliations:** 1 Department of Pharmacology and Physiology, Faculty of Medicine, University of Zaragoza, C/Domingo Miral s/n, 50009, Zaragoza, Spain; E-Mails: tarodmaul@hotmail.com (S.M.-P.); edpiedrafita@yahoo.es (E.P.); jmiana@unizar.es (F.J.M.-M.); lfuentes@unizar.es (L.F.-B.); emb@unizar.es (E.M.-B.); msaenz@unizar.es (M.A.S.); 2 Department of Human Anatomy and Histology, Faculty of Medicine, University of Zaragoza, C/Domingo Miral s/n, 50009, Zaragoza, Spain; E-Mail: lalopicom@hotmail.com

**Keywords:** melatonin, pinoline, indoleamine, free radical, lipid peroxidation, protein oxidation, synaptosome

## Abstract

Since biological membranes are composed of lipids and proteins we tested the *in vitro* antioxidant properties of several indoleamines from the tryptophan metabolic pathway in the pineal gland against oxidative damage to lipids and proteins of synaptosomes isolated from the rat brain. Free radicals were generated by incubation with 0.1 mM FeCl_3_, and 0.1 mM ascorbic acid. Levels of malondialdehyde (MDA) plus 4-hydroxyalkenal (4-HDA), and carbonyl content in the proteins were measured as indices of oxidative damage to lipids and proteins, respectively. Pinoline was the most powerful antioxidant evaluated, with melatonin, *N*-acetylserotonin, 5-hydroxytryptophan, 5-methoxytryptamine, 5-methoxytryptophol, and tryptoline also acting as antioxidants.

## 1. Introduction

Free radicals are atoms or molecules with one or more unpaired electrons in their outer orbital, which makes them unstable, highly reactive species. They are capable of attacking any neighboring molecules by abstracting electrons, thereby re-establishing electronic stability [1]. When free radicals react with phospholipids of biological membranes, they initiate a chain reaction, known as lipid peroxidation, which results in loss of membrane-dependent functions and even cellular death [2]. It has been proposed that free radicals alter the structure of proteins and thus contribute to cellular aging [3]. These modifications include the oxidation of amino acid residue functional groups, cross-linking, and oxidation of the protein backbone, resulting in protein fragmentation [4].

Melatonin is a methoxyindole derivative produced in the pineal gland from the essential amino acid tryptophan in a four-step process, with the enzyme *N*-acetyltransferase being the regulator of melatonin synthesis [5]. Subsequent studies have demonstrated that melatonin is also produced in other tissues such as retina, bone marrow, gastrointestinal tract, gonads and immune system [6–8]. Although melatonin was initially considered a modulator of circadian rhythms and seasonal reproduction in several mammals, extensive research has verified the antioxidant action of melatonin and its beneficial potential in preserving the nervous system from oxidative damage [9]. Melatonin efficiently protects biomolecules, including lipids, proteins and nucleic acids [10] and preserves membrane fluidity [11]. All these positive effects of melatonin may be related to its ability to scavenge reactive oxygen species including hydroxyl (•OH), superoxide anion (O_2_^•−^), and peroxyl radicals (ROO•), singlet oxygen (^1^O_2_), nitric oxide (•NO), peroxynitrite anion (ONOO^−^) and hypochlorous acid (HClO) [12–17], to stimulate antioxidant enzymes such as superoxide dismutase, glutathione peroxidase and catalase [18,19], and to inhibit some prooxidant enzymes, e.g., nitric oxide and aminolevulinate synthases [18,20].

The nervous system has an elevated blood flow and metabolic rate in mammals. Nerve tissue is highly sensitive to free radicals for several main reasons: firstly, the nervous system has a poorly developed endogenous antioxidant defense system, secondly, in the membranes of the nervous tissues, lipids are rich in polyunsaturated fatty acids, which are particularly sensitive as substrates in the lipid peroxidation reaction; and thirdly, certain areas of the brain produce hydrogen peroxide and they are rich in iron which may contribute to generating •OH *via* the Fenton reaction [9,21,22].

While numerous studies have shown the ability of melatonin to achieve protection against free radical damage to the central nervous system, there is little information regarding the antioxidant ability of other pineal indoleamines and β-carbolines synthesized in the pineal gland. In the current study, we have investigated and compared the protective effects of melatonin and structurally-related compounds in preventing oxidative damage to lipids and proteins in membranes isolated from the whole brain. The *in vitro* model for induction of radical species *via* the Fenton reaction used a combination of FeCl_3_ plus ascorbic acid, which is widely accepted in the field of oxidative stress research [23,24]. Lipid peroxidation was assessed by measuring the concentrations of malondialdehyde (MDA) and 4-hydroxyalkenals (4-HDA) and protein oxidation was monitored using methods based on carbonylation.

## 2. Results and Discussion

### 2.1. Time Kinetics of Oxidation in the Synaptosomes

**Figure 1.**
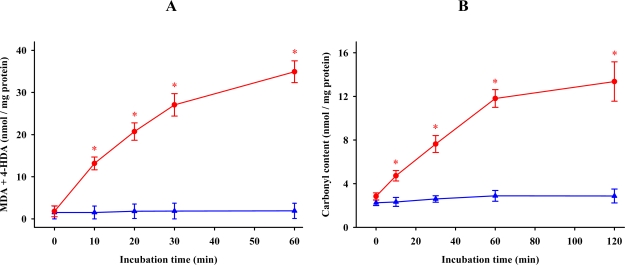
Changes in the levels of lipid (A) and protein (B) oxidations as a function of time in synaptosomal membranes incubated at 37 °C in the presence (
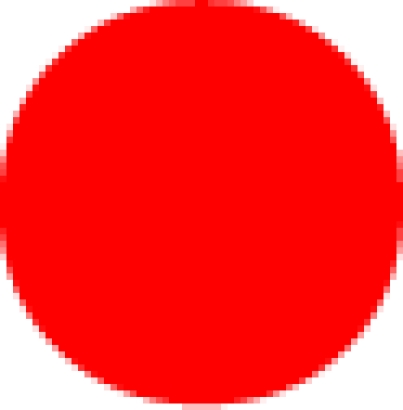
) or absence (
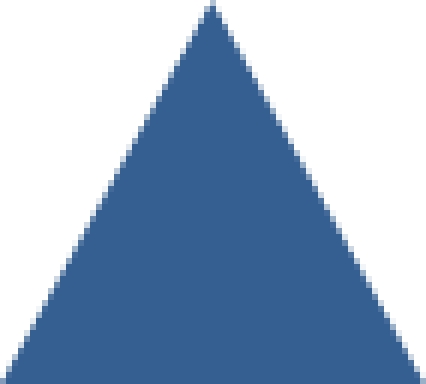
) of 0.1 mM FeCl_3_ and 0.1 mM ascorbic acid. Results are given as means ± SE (n = 6). * *P* < 0.05, compared to controls at the same time.

Incubation of synaptosomes in the absence of iron and ascorbic acid did not modify MDA + 4-HDA levels and synaptosomal protein carbonylation. However, the addition of 0.1 mM FeCl_3_ and ascorbic acid to the incubation medium resulted in the elevation of these indices in a time-dependent manner. Lipid peroxidation increased steadily during the first 30 min of the incubation period (Figure 1A). Since no statistically significant difference occurred between the 30 and 60 min measurements, we selected a 30 min incubation period for the subsequent studies. Similarly, protein carbonylation increased over the first 60 min and then reached a constant value (Figure 1B). Therefore, we conducted all additional studies for a time of 60 min.

### 2.2. Tryptophan and 5-hydroxytryptophan

**Figure 2.**
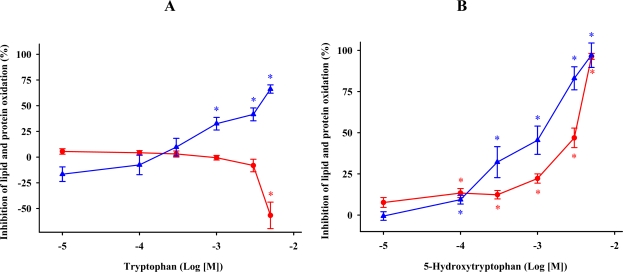
The effects of tryptophan (A) or 5-hydroxy-tryptophan (B) concentrations on FeCl_3_ and ascorbic acid-induced lipid (
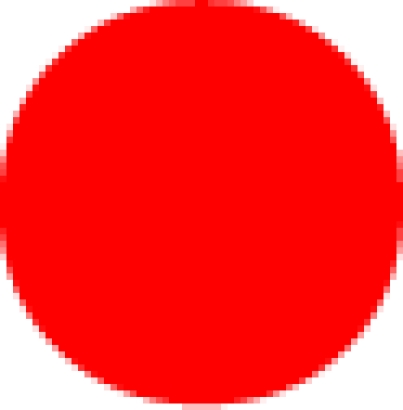
) and protein (
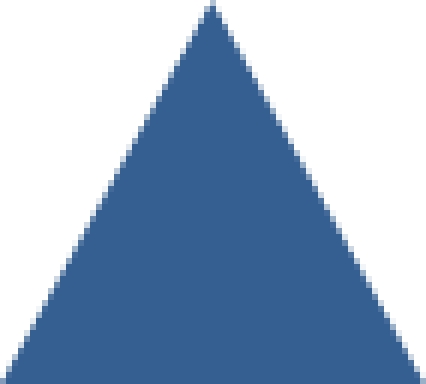
) oxidations in synaptosomes isolated from rat brains. The incubation time was 30 min for lipid peroxidation and 1 h for protein oxidation. Both indoleamines are expressed as the logarithm of its molar concentration. Results are given as means ± SE (n = 6) and are expressed as a percentage of control membrane preparations. * *P* < 0.05 *vs*. membranes treated only with FeCl_3_ and ascorbic acid.

We examined the effects of tryptophan, a physiological constituent of the proteins, on lipid and protein oxidation due to iron and ascorbic acid (Figure 2A). Tryptophan, at concentrations of ≥3 mM, significantly increased levels of MDA + 4-HDA in the membrane suspension. Exposure of the membrane suspension to 1 mM or higher tryptophan significantly reduced protein oxidation (Figure 2A). We have recently shown that 0.01–3 mM tryptophan failed to preserve the fluidity of hepatic membranes from the rigidity induced by lipid peroxidation [25].

Consistent with our results, tryptophan promoted oxidative stress in the cerebral cortex of rats [26]. Moreover, in rats fed with tryptophan-supplemented diets, the amino acid appeared to enhance lipid peroxidation in plasma as well as several tissues [27,28]. On the other hand, tryptophan showed a weak antioxidant behavior which prevented the formation of the 2,2′-azino-bis(3-ethylbenz-thiazoline-6-sulfonic acid) (ABTS) cation radical by scavenging •OH and ROO• that was five orders of magnitude lower than melatonin [29]. The effect of tryptophan on protein oxidation agree with *in vivo* studies that have claimed that tryptophan reduces edema and oxidative stress in cerulein- or ischemia/reperfusion-induced pancreatitis [30] and accelerated the healing of gastric ulcers induced by administration of acetic acid, ethanol, and aspirin [31,32].

5-Hydroxytryptophan is biosynthesized from tryptophan by 5-tryptophan hydroxylase in the pineal gland. 5-Hydroxytryptophan prevented lipid and protein oxidations in a concentration-dependent manner (Figure 2B). These results are consistent with two previous studies showing that 5-hydroxytryptophan reduced lipid peroxidation and reversed membrane rigidity in hepatic cells and microsomal membranes treated with iron [23,25]. It has been proposed that the indole nucleus of tryptophan is the moiety of the molecule responsible for the antioxidant function [33]. Although our study did not address the chemical mechanism of the antioxidant activity, according to our results it seems reasonable to consider that hydroxylation of the amino acid activates its antioxidant behavior, which may be related to its ability to transfer electrons to free radicals.

### 2.3. Methoxytryptamine, N-acetylserotonin, and Tryptamine

**Figure 3.**
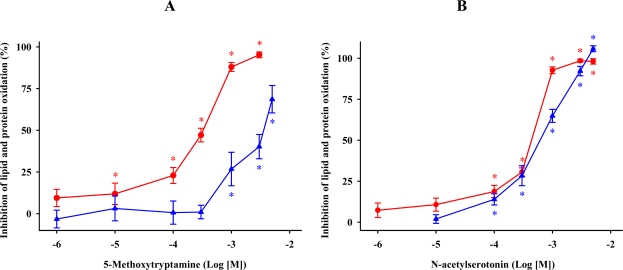

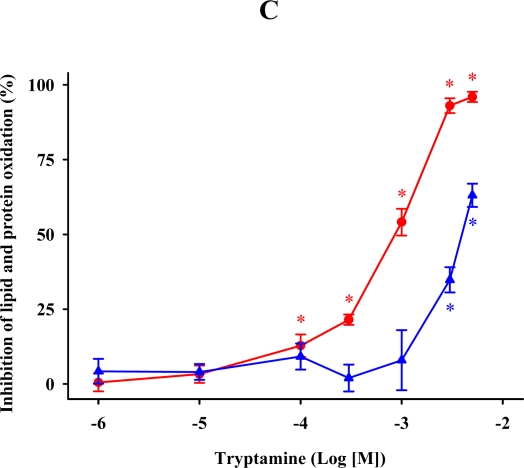
Percent-inhibition curves of concentrations of 5-methoxytryptamine (A), *N*-acetylserotonin (B), and tryptamine (C) in reducing FeCl_3_ and ascorbic acid-induced lipid (
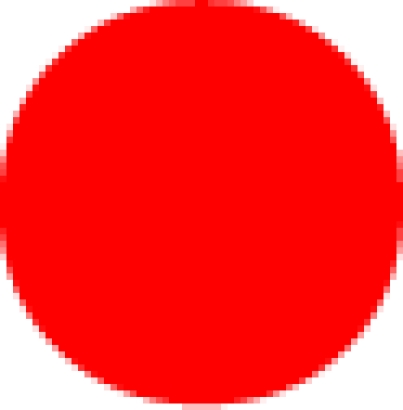
) and protein (
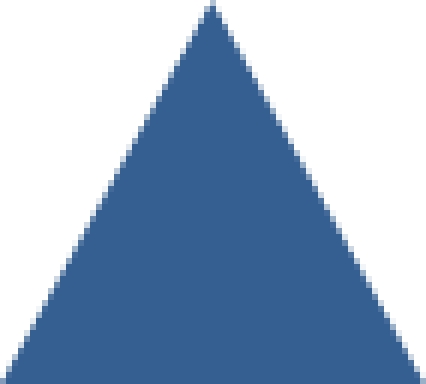
) oxidations in synaptosomal membranes. 5-methoxytryptamine, *N*-acetylserotonin and tryptamine are expressed as a logarithm of its molar concentration. Results are given as means ± SE (n = 6) and are normalized with respect to control membrane preparations that were not exposed to oxidative conditions. * *P* < 0.05 *vs*. membranes treated only with FeCl_3_ and ascorbic acid.

The ability of 5-methoxytryptamine, *N*-acetylserotonin and tryptamine to inhibit MDA + 4-HDA formation and to augment the carbonyl content in the proteins of synaptosomal membranes treated with FeCl_3_ and ascorbic acid was concentration-dependent (Figure 3). Tan and colleagues reported the first observation of the antioxidant effect of 5-methoxytryptamine [12]. They demonstrated that this indoleamine efficiently scavenged •OH produced by photolytic rupture of H_2_O_2_. Subsequent reports also showed that 5-methoxytryptamine reduced lipid peroxidation induced by FeSO_4_ and carbon tetrachloride in biomembranes as well as a variety of homogenates from several tissues [34–36]. An *in vivo* study reported that 5-methoxytryptamine prevented lipid peroxidation and the decrease in glutathione concentrations in active muscles of Sprague-Dawley rats after a period of acute exercise [37].

*N*-Acetylserotonin has been implicated in the acceleration of the pro-oxidant activity of ascorbate [38] and its antioxidant role has been supported by several *in vitro* studies which showed that *N*-acetylserotonin decreased the oxidation in low-density lipoproteins caused by copper [39,40], protected DNA against harmful exposure to chromium III and hydrogen peroxide [41], and reduced lipid peroxidation due to iron, hydrogen peroxide and 2,2′-azobis(2-amidinopropane) [34,42–45]. In addition, *N*-acetylserotonin prevented the formation of ultraviolet-radiation-induced cataracts in rats [46] as well as hepatic lipid peroxidation in rats treated with the toxin α-naphthylisothiocyanate, although in this study the indoleamine failed to prevent increases in hepatic enzymes and bilirubin levels in serum [47].

Little is known about the antioxidant ability of tryptamine. This molecule reduced the formation of thiobarbituric acid reactive substances (TBARS) induced by *tert*-butyl hydroperoxide, the hyperglycemia subsequent to treatment with alloxan [48], and the nitration of the amino acid tyrosine caused by ONOO^−^ [49]. All these results and our data are in agreement with the observation of Poeggeler *et al.* [29] who calculated the reaction rates of tryptamine in scavenging •OH and ROO• as 0.80 × 10^6^ and 0.96 × 10^6^ mol·L^−1^·s^−1^, respectively.

Under our experimental conditions, we determined the concentrations of a variety of compounds that reduced lipid peroxidation and protein carbonylation by 50% (IC_50_) (Table 1). Among these compounds, the lowest IC_50_ value for blocking lipid peroxidation was calculated for 5-methoxytryptamine. However, in terms of protein carbonylation, the IC_50_ values showed that *N*-acetylserotonin was more active than 5-methoxytryptamine and tryptamine. Thus, it seems reasonable to deduce that tryptamine is a molecule with relatively poor antioxidant capacity when compared to 5-methoxytryptamine or *N*-acetylserotonin. The substitution of a hydrogen atom for a methoxy group in position 5 of the tryptamine activates its antioxidant behavior, as reflected by a reduction of the IC_50s_ required to prevent lipid peroxidation (62.1%) and protein carbonylation (14.1%). *N*-Acetylation of tryptamine and the incorporation of a hydroxyl group in C_5_ of the indole ring also enhanced the antioxidant ability of tryptoline in preventing lipid and protein oxidation by 50.0 and 83.6%, respectively. These results suggest that methoxylation, hydroxylation, and *N*-acetylation of tryptamine reinforced its protective effect against free radical damage, possibly through mechanisms mediated by an improvement of the ability to donate electrons as well as modifying the solubility of tryptamine in the incubation medium, which would give greater access of the indoleamine to the free radicals.

### 2.4. Melatonin

**Figure 4.**
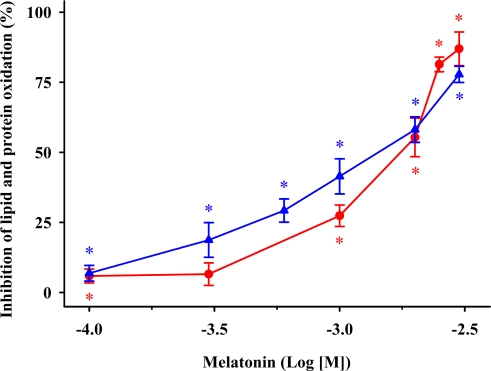
The ability of melatonin to decrease synaptosomal lipid and protein oxidations. (
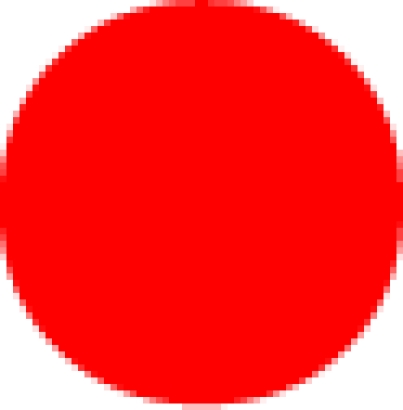
) MDA + 4-HDA concentrations; (
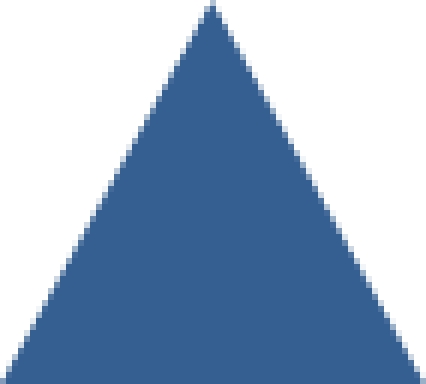
) carbonyl contents. Melatonin is expressed as the logarithm of its molar concentration. Values are means ± SE (n = 6) and are expressed relative to comparable control membrane preparations. * *P* < 0.05 *vs.* membranes treated exclusively with FeCl_3_ and ascorbic acid.

Melatonin’s inhibitory effects of iron- and ascorbic acid-induced lipid and protein oxidations are concentration-dependent (Figure 4). Melatonin at 3 mM prevented both oxidative indices almost completely; in this case, no significant differences in MDA + 4-HDA concentration and carbonyl contents were observed relative to control membranes without FeCl_3_ and ascorbic acid.

The efficiency of melatonin as an antioxidant has been demonstrated in numerous studies [9]. In the brain, melatonin prevented lipid peroxidation under various experimental conditions. Thus, this indoleamine reversed the prooxidant effects caused by the excitatory amino acid glutamate [50], kainic, quinolenic, and okadaic acids [51–53], Alzheimer amyloid peptide [54], 1-methyl-4-phenyl-1,2,3,6-tetrahydropyridine [55], homocysteine [56], and bacterial lipopolysaccharide [57]. Melatonin also reduced the severity of ischemia-reperfusion injury in brain [58]. Tan and colleagues [12] reported that melatonin is a very efficient •OH scavenger, while, in a exhaustive study extended to a wide number of antioxidants, Poeggeler *et al.* [29] claimed that melatonin had an elevated reaction rate with •OH and ROO•. Melatonin’s ability to reduce lipid peroxidation is assumed to be related, at least partially, to its direct scavenging activity. However, several other mechanisms may be involved in its protective effects against these neurotoxins, e.g., by stabilizing cell membranes, allowing them to resist effectively free radical toxicity [11], and by stimulating antioxidative enzymes [59].

Previous *in vitro* evidence indicates that melatonin protects synaptosomal membranes against lipid peroxidation mediated by aluminum and sodium nitroprussiate [24,60,61]. The reduction of carbonylation of proteins by melatonin was described for the first time by Kim *et al.* [62], who proved that melatonin reduced the formation of carbonyl groups in a solution of bovine serum albumin treated with ascorbate-Fe^3+^-EDTA. Daily administration of melatonin in senescence-accelerated mice reduces protein carbonylation in the cerebral cortex [63]. These findings are consistent with the results of the current study wherein melatonin decreased the accumulation of MDA + 4-HDA levels and protein carbonylation in synaptosomes incubated with 0.1 mM FeCl_3_ and 0.1 mM ascorbic acid.

### 2.5. 5-Methoxytryptophol and 5-methoxy-3-indoleacetic Acid

**Figure 5.**
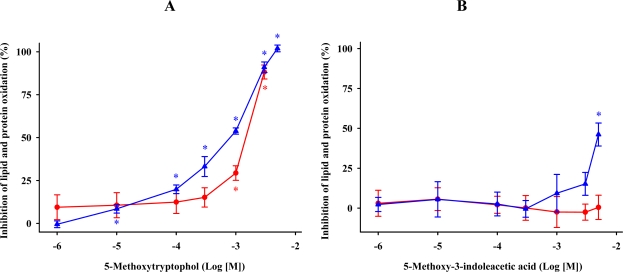
Inhibition of lipid (
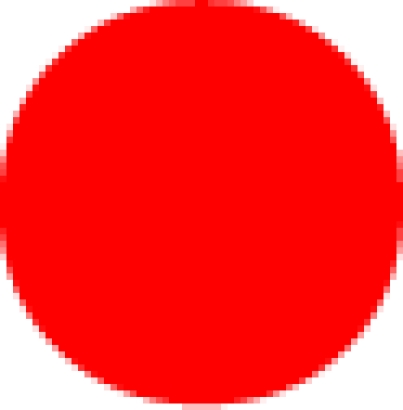
) and protein (
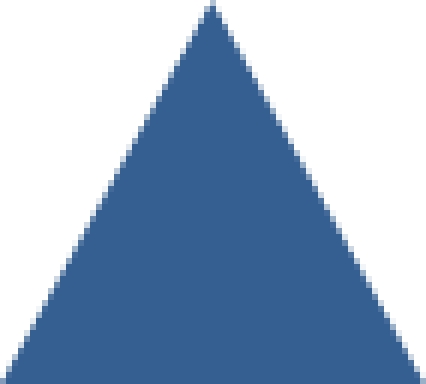
) oxidation by 5-methoxytryptophol (A) or 5-methoxy-3-indoleacetic acid (B) induced by FeCl_3_ and ascorbic acid. The concentrations of these indoleamines are expressed as the decimal logarithm of its molar concentration. Percentage inhibitions are shown as means ± SE (n = 6) and are expressed relative to control membrane preparations. * *P* < 0.05 *vs* membranes treated only with FeCl_3_ and ascorbic acid.

Biosynthesis of 5-methoxytryptophol and 5-methoxy-3-indoleacetic acid occurs from serotonin and is catalyzed by monoamine oxidase and hydroxyindole-*O*-methyltransferase [64]. 5-Methoxytryptophol inhibited FeCl_3_ and ascorbic acid-induced lipid and protein oxidation in a dose-dependent manner (Figure 5A). The minimal effective concentration of 5-methoxytryptophol that significantly reduced oxidative damage was 0.1 mM (*P* ≤ 0.05). All concentrations of 5-methoxytryptophol greater than 0.5 mM caused progressively greater reductions in MDA + 4-HDA levels and protein carbonylation in synaptosomal membranes. However, 5-methoxy-3-indoleacetic acid failed to protect the membranes in terms of biochemical markers of oxidative stress. Only high levels (5 mM) of 5-methoxy-3-indoleacetic acid reduced the synaptosomal carbonylation by 46.1% (Figure 5B).

Under our experimental conditions, 5-methoxytryptophol showed an antioxidant action. Although limited knowledge exists on the antioxidant capacity of this indoleamine, our data is in agreement with previous reports that demonstrate that 5-methoxytryptophol reduced the formation of the ABTS cation radical acting as a potent electron donor [29], decreased iron-induced hepatic microsomal membrane rigidity [65], and prevented lipid peroxidation due to hydrogen peroxide in rat brain homogenates [66]. Earlier studies with 5-methoxy-3-indoleacetic acid claimed that this molecule was unable to prevent the oxidation of low density lipoprotein [36]. However, the 5-methoxy-3-indoleacetic acid reaction rate with •OH and ROO•, exclusively when the incubation medium had a very low pH [29]. The difference in pH in the incubation medium may explain the lack of antioxidant effect of 5-methoxy-3-indoleacetic acid in our study.

### 2.6. β-Carbolines

**Figure 6.**
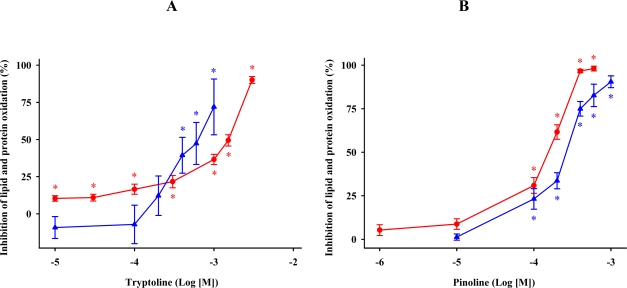
Response of tryptoline (A) or pinoline (B) in preserving synaptosomes from lipid (
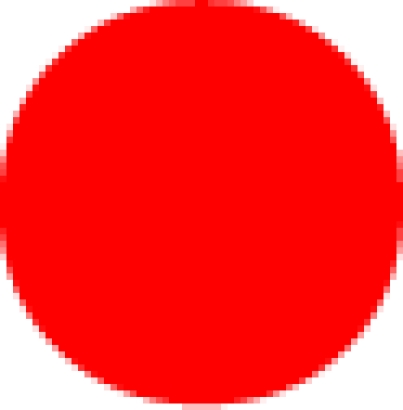
) and protein (
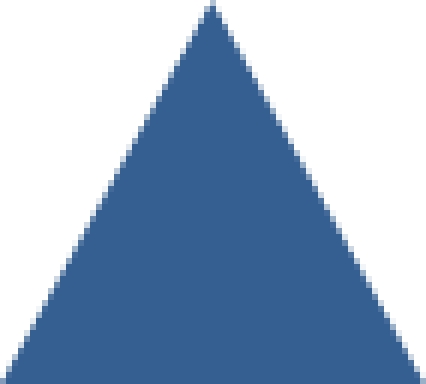
) oxidation after oxidative stress. The concentrations of these β-carbolines are expressed as the decimal logarithm of its molar concentration. Values are means ± SE (n = 6) and are expressed relative to control synaptosome preparations. * *P* < 0.05 *vs* membranes treated exclusively with FeCl_3_ and ascorbic acid.

β-carboline formation has been proposed via the Pictet-Spengler reaction by condensation of indoleamines with aldehydes [64,67]. We observed that progressively increasing concentrations of tryptoline or pinoline prevented MDA + 4-HDA formation and protein carbonylation in a concentration-dependent manner (Figure 6). Pinoline proved to be more potent than tryptoline in reducing lipid and protein oxidation. These data agree with a previous study showing that pinoline was more active in reducing lipid peroxidation in brain homogenates exposed to hydrogen peroxide [68]. Tryptoline differs from pinoline in the absence of the methoxy group. It has been proposed that methoxylated tryptophan derivatives are more effective antioxidants than hydroxylated compounds [12,29]. The data reported here for β-carbolines is consistent with this observation. Several reports have claimed that pinoline reduced lipid peroxidation induced by hydrogen peroxide, iron, sodium nitroprussiate, copper (I) iodide, aluminum, and glutamate [24,44,60,68–71]. Herein, we show for the first time that tryptoline also reduces oxidative stress in synaptosomal membranes. As for pinoline, our data are in agreement with two previous studies stating that pinoline limits oxidative damage to proteins in synaptosomes [24,60].

## 3. Experimental Section

### 3.1. Chemicals

All chemical reagents were of the purest available quality obtainable from commercial sources. FeCl_3_, ascorbic acid, melatonin, tryptophan, 5-hydroxytryptophan, 5-metoxytryptamine, *N*-acetyl-serotonin, tryptamine, melatonin, 5-methoxy-3-indoleacetic acid, 5-methoxytryptophol, tryptoline, and pinoline were purchased from Sigma-Aldrich (Madrid, Spain). The Bioxytech LPO-586 kit for lipid peroxidation was obtained from Cayman Chemical (Ann Arbor, MI, USA). Melatonin was diluted in absolute ethanol and Tris-HCl 50 mM, with ethanol constituting 2% (v/v) of the final solution. Tryptamine, 5-methoxytryptamine, 5-methoxytryptophol, 5-methoxy-3-indoleacetic acid, and pinoline were diluted in methanol and incubation buffer; the concentration of methanol was 2% (v/v) in the final solution. The rest of the reagents were dissolved in incubation buffer. All solutions were freshly prepared just prior to use.

### 3.2. Animals and Isolation of Synaptosomes

Fifty Sprague-Dawley male rats, weighing 200–250 g were purchased from Harlan-Ibérica (Barcelona, Spain). Before being sacrificed, they were acclimated for two weeks, three animals in each Plexiglas cage. The brains were quickly removed, washed in cold saline solution (0.9% NaCl), and homogenized 1/10 (w/v) in 0.32 M sucrose using a motor-driven Teflon homogenizer. Membranes were isolated following the protocol previously described by Millán-Plano and co-workers [24]. Briefly, tissue homogenates were centrifuged at 1,000 × g at 4 °C for 10 min to remove the nuclei and cellular debris. Then, supernatants were centrifuged at 30,000 × g for 20 min at 4 °C and the resulting pellets were resuspended in H_2_O (1/10, v/v), homogenized, and centrifuged at 10,000 × g for 20 min at 4 °C. Following this centrifugation, supernatant and buffy coat was removed, homogenized, and then recentrifuged at 48,000 × g at 4 °C for 20 min The pellet was washed twice and the final pellet was resuspended 1/2 (v/v) in Tris-HCl buffer (pH = 7.4), and frozen at −80 °C until assay.

### 3.3. Experimental Design

The induction of oxidative stress to the synaptosomal membranes was performed by using a •OH generator system. This system was based on the models proposed by Wong *et al.* [72] and Sahu and Washington [73] using FeCl_3_ and ascorbic acid as prooxidative reagents. Aliquots of synaptosomal membranes, 0.5 mg/mL, were suspended in Tris-HCl 50 mM buffer and incubated in a water bath with shaking at 37 °C with or without 0.1 mM FeCl_3_, 0.1 mM ascorbic acid and in the absence or presence of the indoleamines (0.001–5 mM). The duration of the incubation was determined by time kinetics, measuring the formation of MDA + 4-HDA and carbonyl groups over 120 min after initiating incubation. The oxidation of the membranes was quenched by adding 2 mM EDTA and by placing the membrane solution into ice-cold water for 10 min. Control synaptosomes and those with induced oxidation were exposed to the same conditions (incubation and addition of ethanol or methanol) as the samples treated with the indoleamines.

### 3.4. Analytical Procedures

MDA + 4-HDA levels were used as an index of peroxidation of the synaptosomal lipids [74]. The levels of the lipid peroxidation products were measured using the Bioxytech LPO-586 kit. In this assay, MDA and 4-HDA react with *N*-methyl-2-phenylindole to yield a stable chromophore with a peak of maximum absorbance at 586 nm. Results are expressed as nmol MDA + 4-HDA/mg of synaptosomal protein. The protein concentrations were determined employing the method of Lowry [75], using bovine serum albumin as the standard.

Carbonyl groups in the synaptosomal proteins were measured using the reaction with 2,4-dinitrophenylhydrazine (DNPH) described by Levine *et al*. [76], with slight modifications. Aliquots (100 μL) of 50 mM Tris-HCl buffer and 200 μL of 10 mM DNPH solution were added to 1 mL of synaptosomal membranes, and the mixture was incubated al 37 °C for 1 h. Aliquots (325 μL) of 50% ice-cold trichloroacetic acid was added to the mixture and the samples were placed on ice for 10 min. The pellets obtained after centrifugation at 3,000 × g for 10 min were washed three times with ethanol/ethyl acetate (1:1, v/v). The final pellets after the third washing were dissolved in 6 M guanidine and incubated at 37 °C for 15 min. After centrifugation at 12,000 × g for 10 min, the absorbance of the supernatants was measured spectrophotometrically at 375 nm. Protein carbonyl groups were estimated by using the molar absorption coefficient of 22,000 M^−1^·cm^−1^ for DNPH derivatives, and its concentration was expressed as nmol carbonyl groups/mg protein. Guanidine solution was used as a blank.

### 3.5. Statistical Analysis

All results are expressed as means ± standard error of at least five independent experiments. Student’s *t*-tests were used for comparison of the means. Differences were accepted as being statistically significant when *P* < 0.05.

## 4. Conclusions

The findings presented in this paper demonstrate that melatonin and other structurally-related compounds afford protection against FeCl_3_ and ascorbic acid-induced lipid and protein oxidation in synaptosomes isolated from rat brain and reinforce the idea that several secretory products of the pineal gland may play a role in protecting biological membranes from oxidative damage. Table 1 summarizes the concentrations of these molecules required to reduce lipid and protein oxidations by 50% (IC_50_). Although these results suggest that pinoline is the most powerful antioxidant evaluated under our *in vitro* experimental conditions, since there is no data regarding the *in vivo* effects of β-carbolines, further studies should be conducted to define the antioxidant properties of these molecules. Regarding the indoleamines, while ample evidence demonstrates that melatonin achieves neuroprotection *in vivo* against free radical damage in a range of toxicological models [9,10], further studies are needed to verify the antioxidant behavior of other indoleamines. When melatonin was compared *in vivo* to other indoleamines, melatonin appeared to be a more active antioxidant and free radical scavenger [47]. Our results are consistent with the idea that several indoleamines and β-carbolines contribute to the antioxidant role of the pineal gland.

## Figures and Tables

**Table 1.** Calculated concentrations (mM) required to inhibit lipid and protein oxidation by a 50% (IC_50_) in preventing lipid and protein oxidation in synaptosomal membranes exposed to FeCl_3_ and ascorbic acid.

**Substance (n = 6)**	**Lipid peroxidation**	**Protein oxidation**
Tryptophan	-	2.92
5-Hydroxytryptophan	3.05	1.01
5-Methoxytryptamine	0.31	3.40
*N*-acetylserotonin	0.42	0.65
Tryptamine	0.84	3.96
Melatonin	1.75	1.40
5-Methoxy-3-indoleacetic acid	-	-
5-Methoxytryptophol	1.75	0.76
Tryptoline	1.49	0.63
Pinoline	0.15	0.25
